# The oral microbiome profile of Pakistani infants characterized by 16S rRNA amplicon sequencing

**DOI:** 10.1016/j.dib.2026.112449

**Published:** 2026-01-07

**Authors:** Muhammad Shahzad, Muhammad Ismail, Muhammad Junaid ul Islam, Sarfaraz Yumna, Taj Irum, Khan Malalai, Israr Sara, Ziad Al Nabhani, Simon C Andrews

**Affiliations:** a Faculty of Dentistry, Zarqa University, Jordan; b Institute of Basic Medical Sciences, Khyber Medical University Peshawar, Pakistan; c Department of Visceral Surgery and Medicine, Bern University Hospital, Bern, Switzerland; d Maurice Müller Laboratories, Department for Biomedical Research, University of Bern, Bern, Switzerland; e School of Biological Sciences, Health and Life Sciences Building, University of Reading, Reading RG6 6EX, United Kingdom

**Keywords:** Oral microbiome, Infants, Pakistan, Malnutrition, Cohort

## Abstract

The oral microbiome is the second most complex and diverse ecosystem in the human body. A number of longitudinal studies assessing oral microbiome development in diverse populations has been reported recently. However, oral microbiome development in vulnerable populations such as infants who are at risk of malnutrition is rarely explored. The current study aims to assess oral bacterial community development and associated factors in Pakistani infants residing in malnutrition endemic areas of Pakistan. Data and oral swab samples were collected from infants (*n* = 71) at baseline (age <28 days) and 3-months follow-up (*n* = 65) followed by DNA extraction, PCR amplification and 16S rRNA amplicon sequencing on a DNBSEQ-G400 platform. Of the total 136 samples, 119 samples were successfully sequenced and analyzed further. Bioinformatics and statistical analyses were performed using Cutadapt, FLASH and R. Overall, the *Bacillota* (formerly known as *Firmicutes*) was the predominant bacterial phylum, accounting for 87.6 % relative abundance at baseline and 84.3 % at 3-months. The *Streptococci* and *Veillonella* were the predominant bacterial genera with 66.9 % and 13.4 % relative abundance at baseline and 55.4 % and 26.1 % at 3-months, respectively.

This study provides the first comprehensive insights into oral bacterial community development of vulnerable infants at risk of malnutrition. The data can be used to longitudinally assess oral microbiome develop during early infancy and associated maternal, infant and environmental factors. Sequencing data are deposited in the NCBI Sequence Read Archive as BioProject PRJNA1303979.

Specifications Table

**Table:** 

Subject	Microbiology.
Specific subject area	Metagenomic (Human Oral Microbiome)*.*
Type of data	Table, Raw, Analysed
Data collection	The current study is embedded in a longitudinal cohort (the CHAMP study) conducted in Khyber Pakhtunkhwa province of Pakistan [1]. Household sociodemographic characteristics and oral swab samples were collected from newborn infants at baseline (*n* = 71) and 3-months (*n* = 65). DNA was extracted MagPure DNA KF Kit B (MAGEN, Guangzhou, China) followed by 16S rRNA amplicon sequencing on DNBSEQ-G400 platform (BGI-Shenzhen, China).
Data source location	The samples were collected in District Swat, Pakistan. Latitude and Longitude: 35.0848° N, 72.2334° E
Data accessibility	Repository name: NCBI Sequence Read Archive (SRA) Data identification number: BioProject PRJNA1303979 Direct URL to data: https://dataview.ncbi.nlm.nih.gov/object/PRJNA1303979?reviewer=34iirk8si4vbh5l11elagqha3e
Related research article	*None.*

## 1 Value of the Data

• The dataset explores early (first 3 months) oral bacterial community development in Pakistani infants residing in malnutrition endemic areas of Pakistan.
• The data provides deeper insights into the role of maternal and infants dietary intake and nutritional status on oral bacterial community development.
• The longitudinal nature of the research will help to establish causal relationships between nutritional status and oral microbiota composition, and provide stronger evidence on how malnutrition drives oral microbiota dysbiosis.
• The data contributes to global understanding of oral microbiota development in infants, especially those residing in low- and middle-income countries, and offers novel insights into using oral microbiota as diagnostic tool for early health risk assessment.

## 2 Background

The oral microbiota, encompassing >700 different species, plays a key role in oral and systemic health [2]. Acquisition and development of the oral microbiota begin immediately after birth and evolve from a sparse, pioneer community into a more complex, stable and mature ecosystem during infancy and childhood [3,4]. At birth, the oral cavity is mainly colonized by maternal and environmental bacterial taxa, e.g. Streptococcus salivarius, Streptococcus mitis, and Veillonella species, and is influenced by factors such as delivery mode and antibiotic use. During infancy, the feeding practices, i.e. whether the baby is breastfed or formula fed, greatly influence oral microbiome diversity and composition. Tooth eruption and introduction of solid foods (weaning) marks the next major shift in oral microbiome composition which is characterised by the emergence of biofilm formers (e.g. Streptococcus sanguinis, Streptococcus gordonii) and anaerobic bacterial species (e.g. Fusobacterium, Prevotella). By late childhood, the oral microbiome gradually matures into a more stable, adult like microbiome characterised by high relative abundance of Streptococcus, Veillonella, Neisseria and Actinomyces species.

Until now, longitudinal studies characterising oral microbiome succession and development have been mainly conducted in high income and developed countries of Europe, East Asia and the Amiericas [5]. However, comparable research in the South Asian countries remains critically scare despite the region bearing the highest burden of oral diseases in the world [6]. Previous studies have primarily focused on oral diseases in adults and gut microbiome development in infants. Furthermore, oral microbiota development in infants from high-risk population, especially those residing in malnutrition endemic areas, is rarely explored.

Malnutrition is a global public health issue, especially in developing countries such as Pakistan [7]. The consequences of malnutrition are devastating, especially the increase in morbidity and mortality in children under five years of age [8,9]. Malnutrition during the first 1000 days of life can also significantly impact gut microbiota development including alteration in microbial diversity and functions[10]. This phenomenon, also known as microbial dysbiosis, leads to alterations in immune development in children, and enhanced susceptibility to infection and non-communicable diseases in later life[11]. Similarly, infants born in regions with high prevalence of malnutrition are also prone to develop oral microbiota dysbiosis, and associated oral and systemic diseases and condition. However, research to date is limited and insufficient to fully characterise the relationship between the oral microbiome and malnutrition in early infancy. The current study aims to longitudinally assess oral bacterial community development in Pakistani infants at 1 and 3 months of age, who are born in areas where malnutrition is endemic.

## 3 Data Description

**Fig. 1:**
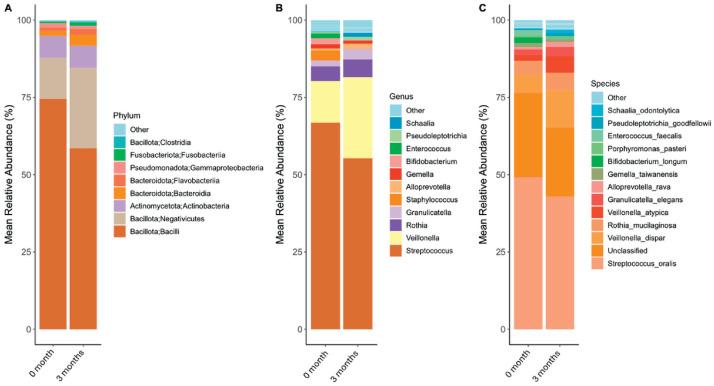
Relative abundance of the major bacterial taxa over time showing progressive changes in microbiota composition at (a) phylum, (b) genus and (c) species level.

The data presented here represent oral microbiota development of newborn infants from District Swat, Pakistan. A total of 136 oral swab samples were collected from infants at baseline (<28 days; *n* = 71) and at 3-months follow-up (*n* = 65). These were subjected to DNA extraction, PCR amplification of the V3–V4 hypervariable region of 16S rRNA followed by 16S rRNA amplicon sequencing on a DNBSEQ-G400 platform (BGI-Shenzhen, China). Of these, 17 samples failed to qualify for the library preparation stage and were therefore excluded from further analysis. Taxonomic analysis indicated a diverse oral microbiota composition with changes in the diversity and relative abundance of key bacterial taxa between the two time points. Fig. 1A-C present the relative abundance of bacteria at phylum, genus and species level, at baseline (<28 days) and 3-months for oral swab samples.

Overall, 34 different phyla were identified in the oral microbiome of infants. *Bacilli* were the most abundant bacterial phylum followed by *Negativicutes* and *Actinobacteria*, at both time points. However, their relative abundance was different between the 1 and 3 month samples. For example, the relative abundance of *Bacilli* was 74.7 % at baseline while at 3-months, their relative abundance decreased to 58.9 %. In contrast, the relative abundance of *Negativicutes* increased from 12.9 % at 1-month to 25.4 % at 3 months. A total of 257 different genera were identified at both time points. *Streptococci* were the most abundant genus with relative abundance of 66.9 % and 55.4 % at baseline and 3-months, respectively. *Veillonella* was the second most abundant genus with higher relative abundance at 3-months (26.1 %) than at baseline (13.4 %). We also identified 355 different bacterial species in the oral microbiota of infants across both time points. Of these, *Streptococcus oralis* was the most abundant bacterial species at both time points. However, the relative abundance was higher (49.1 %) at baseline than at 3-months (42.9 %).

## 4 Experimental Design, Materials and Methods

### 4.1 Study design and population

The present study was nested within a larger cohort study [12] investigating gut microbiome development and associated factors in Pakistani infants during the first two years of life (0–24 months). This sub-study specifically focused on early oral microbiome development (0–3 months) and associated maternal, infant and environmental factors that shape the oral microbiota of infants residing in malnutrition endemic areas of Pakistan. The study site was District Swat, located at 35.0848° N, 72.2334° E in Khyber Pakhtunkhwa province of Pakistan and home to >2.3 million people [13].

Of the total 92 families eligible to participate in the study, 71 mother-infant dyads were successfully recruited in May 2024 for a study that was planned to continue until May 2026 with intervening assessments at a range of time points. The main inclusion criteria for the current study were: healthy infants of any gender; aged 0 – 28 days; and born to parents residing in the CHAMP cohort study site. Children born to underage mothers, or those with oral and systemic disease, or conditions requiring hospitalization, were excluded from the study.

### 4.2 Data collection

Different data were collected from mother and infants at baseline and at the 3-month follow up stage using validated questionnaires. These included data regarding: (a) household demographic and socioeconomic data including assets; (d) health care data of the mother including antenatal and postnatal care data; (c) dietary intake of the data of the mother using validated dietary quality questionnaires [14]; (d) health record data of the infants at baseline and follow-up; and (e) infant and young child feeding practices (IYCF) data. All the data were collected by trained research assistants.

### 4.3 Oral swab sample collection

Oral swab samples from infants were collected at baseline and the 3-months follow up stage using standard methods. For this purpose, the mothers were first instructed to hold the baby secured in a semi-reclined or supine position. Sterile, DNA free oral swabs (Huachenyang Technology, Guangdong, China) were then gently inserted into the infant’s mouth. The target areas (inner cheeks, gums, tongue and hard palate) were swabbed by rotating the cotton end of the swab gently against the desired area. After 15 – 20 s of swabbing, the oral swabs were directly transferred into a sterile collection tube containing sample preservative (Zymobiomics DNA Shield, Zymo Research, California, USA) to protect against sample degradation. The samples were transported to the laboratory within two days at ambient (room) temperature. On arriving at the main laboratory, the samples were stored at −80 °C until further processing.

### 4.4 DNA extraction and 16S rRNA sequencing

DNA was extracted from samples using a MagPure DNA KF Kit B (MAGEN, Guangzhou, China) following the manufacturer instructions. Extracted DNA was quantified using a Nanodrop and the quality was assessed by gel electrophoresis. The mean DNA concentration was 8.2 ± 3.8 ng/µL with an average yield of 0.22 µg per sample. DNA samples were then subjected to PCR amplification of the V3-V4 variable region of the 16S rRNA gene using the primers 338F:ACTCCTACGGGAGGCAGCA and 806R:GGACTACHVGGGTWTCTAAT. The library was prepared using a 2 × Phanta Max Master Mix (VAZYME, China) and subsequently sequenced on a DNBSEQ-G400 platform (BGI-Shenzhen, China)

### 4.5 Bioinformatics analysis

Raw data were filtered to generate high quality clean reads by removing adapter sequences (cutadapt; v.2.6), low quality reads (phred score <20) and ambiguous bases (N base) following standard methods [15]. Barcode sequences were removed from pooling libraries by assigning clean reads to corresponding samples through alignments (0 base mismatch) against barcode sequences by in-house scripts. After quality filtering, a total of 1.5 million reads (average of 0.132 million reads per sample) corresponding to a ConnectRatio 95.4 ± 0.8 indicating the proportion of high-quality reads retained. The average read length was 428 ± 3 bp. Consensus sequences were generated from successful pair-end reads using FLASH (Fast Length Adjustment of Short reads, v1.2.11) [16]. Tags were then clustered into Operational Taxonomic Units (OTUs) with a 97 % similarity threshold using UPARSE (v7.0.1090), with chimeras filtered out using UCHIME (v4.2.40). Representative OTU sequences were taxonomically classified using the RDP Classifier (v2.2) with a confidence threshold of 0.6, aligning against the SILVA reference data base (v138, released Dec 2019). All tags were then mapped back to their corresponding OTU representative sequences using USEARCH_GLOBAL to generate OTU abundance tables for each sample. To minimize biases, sequencing depth was normalized by rarefying all samples to the same read count. The rarefaction depth was chosen based on the sample with the lowest read count but sufficient coverage. Intra-sample dissimilarity index (Alpha diversity) was calculated using the vegan package in R and compared using Wilcoxon rank-sum test. The corresponding p-values were adjusted using the false discovery rate (FDR) method (Benjamini–Hochberg correction). Beta diversity was assessed using Bray–Curtis dissimilarity and visualized via non-NMDS ordination (*k* = 2). Statistical significance in Beta diversity was assessed using PERMANOVA with 999 permutations (adonis2 function).

## Limitations

The study is limited by its relatively small (although not negligible) sample size and absence of a comparison group from areas where malnutrition is not common, such as urban areas of Khyber Pakhtunkhwa. Potential batch effects and sequencing platform limitations (e.g. DNBSEQ generates moderate read lengths compared to long-read technologies such as Oxford Nanopore and PacBio) should also be considered when interpreting the results. Furthermore, the metadata related to participant characteristics and in-depth analysis of factors (maternal, infant and environmental) influencing oral bacterial community development in infants are not included here. These data will be analysed and reported in a separate manuscript currently in preparation.

## Ethics Statement

Ethical approval of the study was obtained from the Ethics Board of Khyber Medical University Pakistan (Ref no DIR/KMU-EB/BR/001–03 dated 11/01/2024). Written informed consent was obtained from the parents/legal guardians of the infants.

## Credit Author Statement

**Muhammad Shahzad:** Conceptualization, Methodology, Funding acquisition, Writing – original draft. **Muhammad Ismail:** Data collection, Data curation, Writing – original draft. **Muhammad Junaid ul Islam:** Data collection, Data curation. **Yumna Sarfaraz:** Data collection, Writing - original draft. **Irum Taj:** Data collection. **Malalai Khan:** Data collection. **Sara Israr:** Data collection. **Ziad Al Nabhani:** Methodology, Writing – review and editing. **Simon C. Andrews:** Supervision, Funding acquisition, Writing – review & editing.

## Data Availability

NCBI SRAOral microbiome development and associated factors among Pakistani infants (Original data).

## Appendix Supplementary materials

Image, application 1
